# Effect of radioactive iodine therapy on hematological parameters in patients with thyroid cancer: systematic review and meta-analysis

**DOI:** 10.3389/fendo.2025.1562851

**Published:** 2025-03-14

**Authors:** Dereje Mengesha Berta, Bisrat Birke Teketelew, Negesse Cherie, Mebratu Tamir, Zufan Yiheyis Abriham, Abiy Ayele Angelo, Amare Mekuanint Tarekegne, Elias Chane, Zewudu Mulatie, Muluken Walle

**Affiliations:** ^1^ Department of Hematology and Immunohematology, School of Biomedical and Laboratory Science, College of Medicine and Health Science, University of Gondar, Gondar, Ethiopia; ^2^ Department of Quality Assurance and Laboratory Management, School of Biomedical and Laboratory Science, College of Medicine and Health Science, University of Gondar, Gondar, Ethiopia; ^3^ Department of Medical Parasitology, School of Biomedical and Laboratory Sciences, College of Medicine and Health Science, University of Gondar, Gondar, Ethiopia; ^4^ Department of Immunology and Molecular Biology, School of Biomedical and Laboratory Science, College of Medicine and Health Sciences, University of Gondar, Gondar, Ethiopia; ^5^ Department of Clinical Chemistry, School of Biomedical and Laboratory Sciences, College of Medicine and Health Sciences, University of Gondar, Gondar, Ethiopia; ^6^ Department of Medical Laboratory Science, College of Medicine and Health Science, Wollo University, Dessie, Ethiopia

**Keywords:** hematological parameters, radioactive iodine, RAI (radioiodine) ablation, therapy, thyroid cancer

## Abstract

**Background:**

Patients with thyroid cancer (TC) are commonly treated with radioactive iodine therapy (RIA) to prevent neoplastic transformation and the re-emergence of cancer cells. However, it has major side effects on blood cells. However, the degree of change in hematological parameters reported varies across studies. Therefore, the aim of this review was to assesses the mean differences in hematological parameters after RAI therapy.

**Methods:**

The relevant articles for this review were identified through extensive searches of databases and the Google search engine. The identified articles were subsequently selected using predetermined eligibility criteria. All relevant information from the screened articles was extracted. The pooled standardized mean differences (SMDs) of the parameters were assessed via a random effects model. The heterogeneity was determined by I^2^ statistics test. Funnel plots and Egger’s test were utilized to assess publication bias.

**Results:**

A total of 17 studies including 4,112 and 3,766 study participants before and after RAI therapy, respectively, were included. The pooled SMDs of the total leucocyte count (TLC) (*10^9^/L) at one, three, six and 12 months and the last follow-up period were 2.39, 2.46, 5.84, 3.19, and 0.53, respectively. Changes in the TLC after one, three and six months of therapy were statistically significant. In terms of the absolute neutrophil count (ANC; *10^9^/L) and absolute lymphocyte count (ALC; *10^9^/L), the pooled SMDs at the last follow-up period were 6.32 and 7.37, respectively. In addition, statistically significant changes in the platelet count (PLT; *10^9^/L) were observed at one, three, six and 12 months and at the last follow-up, with pooled SMDs of 7.01, 0.22, 2.63, 6.61, and 8.76, respectively. Furthermore, statistically significant changes in red blood cells (RBCs; *10^12^/L) and hemoglobin (Hgb; g/dl) were detected after three and six months of therapy, with pooled SMDs of -1.088 and 2.4, respectively.

**Conclusion:**

According to the current systematic review and meta-analysis, radioiodine therapy had a significant effect on hematological parameters. Thus, early screening and correction of hematological toxicity may be helpful for improving quality of life in thyroid cancer patients receiving radioiodine therapy.

**Systematic Review Registration:**

https://www.crd.york.ac.uk/prospero/, identifier CRD42024586449.

## Introduction

Thyroid cancer is the most prevalent type of endocrine cancer that arises from the abnormal growth of epithelial cells in the thyroid gland at the base of the neck (1, 2). Currently, the incidence rate of TC is gradually increasing, accounting for approximately 821,214 new cases and 47,507 deaths globally (3). Its incidence rate is predicted to increase by approximately 30.46% by 2030 (4). However, the incidence rate of TC is high, and TC has a good prognosis (5). Among the subtypes of TC, papillary and follicular thyroid cancers are the most common, whereas medullary and anaplastic thyroid cancers are less common (6). Increasing diagnostic accuracy and surveillance and overdiagnosis are attributable to increasing rates of thyroid cancer (2, 3, 7).

Potential contributing factors of TC are a hereditary history of TC, radiation exposure, exposure to endocrine-disrupting chemicals, long-term exposure to toxins, a history of goiter, and iodine deficiency (1, 6). The diagnosis of TC is based on physical examination, histological examination, imaging studies, blood tests, and identification of genetic abnormalities (1, 8). Thyroid cancer can be treated with surgery, hormone replacement, external bean radiation therapy, and RAI therapy (9). These treatments may be provided in combination based on tumor type and risk stratification (10). Currently, thyroidectomy with subsequent ablation of the RAI is utilized as the standard treatment for differentiated TC, the treatment may be guided based on individual risk assessment and severity (9, 11).

Radioiodine therapy is a medical treatment that involves the administration of RAI isotopes to eliminate thyroid remnants and cancer cells following surgery (12). The removal of thyroid remnants helps prevent neoplastic transformation and the re-emergence of cancer cells (13). However, side effects such as lacrimal duct dysfunction, gastrointestinal dysfunction, pulmonary fibrosis, neck pain, edema, xerostomia, inflammation, nausea, secondary malignancies and hematopoietic system toxicity have been documented (2, 14–16).

Hematopoietic system and other vital organ toxicities are associated with changes in hematological parameters (16–18). Direct damage to blood cells due to the small amount of radioiodine isotopes that enter the blood circulation and tissue affects hematological parameters (2). Induction of cellular apoptosis and suppression of hematopoietic clonogenicity by radiation can also alter the count and function of peripheral blood (19–21). Alterations in genes that are responsible for cell division and DNA repair as a result of the toxic effects of RAI alter the proliferation and differentiation of peripheral blood (2).

Suppression of bone marrow is a major side effect of radioiodine isotopes that adversely affects almost all types of hematological parameters and causes severe hematological toxicity (18). During radioiodine therapy, the radiation emitted affects hematopoietic cells through direct damage and subsequent alterations in hematopoiesis in the bone marrow (21). Additionally, as a result of radioisotopes having greater absorption ability by the bone marrow, they induce damage to DNA and the apoptosis of hematopoietic stem cells (20, 22). On the other hand, gastrointestinal toxicity and subsequent nutritional deficiency are associated with changes in hematological parameters (23, 24). The toxicities of organs such as the thyroid gland, liver and kidney, which play major roles in hematopoiesis by radioisotopes, are also associated with changes in hematological parameters (14, 25).

Alterations in RBCs and their parameters after RAI treatment are related primarily to bone marrow suppression and damage to other vital organs involved in erythropoiesis (14, 26, 27). Structural and functional damage to RBCs due to RAI isotopes that enter directly into the blood circulation and tissue can also be associated with changes in RBCs and their parameters (2). The administration of radioiodine isotopes indirectly changes the oxidative response and induces changes in RBC shape, microcirculation, and life span (2, 26). In addition, the induction of oxidative stress in RBCs causes DNA damage and affects their vascular permeability and mechanical properties (28).

Changes in TLCs and their differences following RAI therapy are a result of organ toxicity and the activation of various inflammatory factors (15, 16, 29). In addition, alterations in growth factors and supportive stroma due to radiation can cause changes in TLCs count and function (29, 30). Induction of cellular apoptosis and alteration of genetic material by radioiodine also affects WBCs (31–33). Like RBCs and TLCs, RAI-associated vital organ damage, apoptosis, and genetic alterations are responsible for changes in the PLT count (15, 20, 21). In addition, direct damage to megakaryocytes and their progenitors by radioisotopes causes changes in PLT number and function (19).

Hematological changes are associated with poor prognosis, increased risk of hospitalization, and other complications (34). The routine evaluation and monitoring of those parameters are beneficial for patients. Most studies reported that common hematological parameters are diminished following RAI therapy (35). However, the reports of studies are not consistent (18, 35–37). Additionally, the degree of hematological changes reported varies. Even though the RAI has a significant effect on hematological parameters and the degree of changes is inconsistent across studies, there is a shortage of systematic reviews and meta-analyses that provide a comprehensive understanding. Thus, the aim of this review was to investigate the pooled mean differences in hematological parameters after RAI therapy in thyroid cancer patients. This may help support clinical decisions and improve patient management.

## Methods

### Protocol registration and design

For this systematic review and meta-analysis, the protocol was initially registered on the International Prospective Register of Systematic Review, and a specific registration number was provided (CRD42024586449). In addition, to estimate the mean differences in hematological parameters after RAI treatment, this systematic review and meta-analysis were designed. Studies conducted to assess changes in hematological parameters after RAI therapy were included in this review. The findings were organized and reported according to the Preferred Reporting Items for Systematic Review and Meta-analysis protocols (PRISMA) (38). The PRISMA checklist was used to ensure that all relevant information was included in the analysis.

### Eligibility criteria

#### Inclusion and exclusion criteria

The literature reporting both the means and standard deviations (SDs) of hematological parameters before and after RAI therapy in thyroid cancer patients was reviewed and included. Besides, prospective and retrospective follow-up studies published in the English language up to October 30, 2024, were included. On the other hand, studies with incomplete or missing outcome data, animal studies, case reports, and studies whose full texts were not available were excluded. Moreover, literature reviews, non-follow-up studies and studies that included other complications affecting hematological parameters were excluded.

### Database and search strategies

Potential articles for this systematic review and meta-analysis were searched via databases such as PubMed, Embase, Medline, the Cochrane Library, Scopus, and Web of Science. Besides, Google Scholar, online archives, and university repository searches were used to add gray literature. The search was conducted by two authors (DMB and MW). The population, intervention, comparator or control and outcome of interest (PICO)-formatted questions were used to conduct a comprehensive search. The search in the related MeSH terms was conjoined with the Boolean operator “OR”; meanwhile, unrelated MeSH terms were conjoined with the Boolean operator “AND” (**Supplementary File 1**).

### Study selection procedure

Two independent authors (DMB and MW) reviewed the identified articles and combined the records into Endnote software version X9 to manage the references. The authors subsequently removed the duplicated records and screened the titles and/or abstracts to include relevant studies. The controversies that occurred between the two authors assigned to screening were resolved through mediation on the basis of predetermined criteria by a third independent reviewer (BBT). The studies eligible after title and abstract screening were selected for further review of the full texts and data extraction.

### Study quality assessment

The quality of all the selected studies was rigorously evaluated via the JBI checklist by two independent authors (DMB and BBT). The evaluation consists of related items such as bias related to temporal precedence, selection and allocation, confounding factors, administration of intervention/exposure, assessment, detection and measurement of the outcome, participant retention, and statistical conclusion validity. A score of N was assigned for no, whereas a score of Y, UC and NA were assigned for yes, unclear and not applicable, respectively (39). After evaluation, only high-quality studies were included (**Supplementary File 2**).

### Data extraction

The Microsoft Excel spreadsheet was formatted with specific information for data extraction and guarantees the accuracy of the data. The information extracted from the studies included the author’s name, year of publication, study setting, sample size before treatment, sample size after treatment, study design, histology of thyroid cancer, stage of thyroid cancer, final follow-up period, mean (SD) hematological parameters before treatment and mean (SD) hematological parameters after treatment for data extraction. All data from the included studies were extracted by one reviewer (DMB) and verified by a second reviewer (BBT).

### Outcome of interest

The outcome of interest was the pooled mean difference in hematological parameters after RAI treatment in patients with thyroid cancer.

### Data synthesis and analysis

Following extraction, the data were exported to STATA 17.0 software for further analysis. The degree of heterogeneity was assessed via the Higgins et al. method. Variability in effect size was estimated via I-square (I^2^) statistics. Hence, the heterogeneity among studies was high, and a random effects model with 95% confidence intervals was utilized to determine the standard mean difference (SMD). Forest plots were constructed on the basis of the source to present pooled estimates of the SMD. Subgroup analysis was conducted to assess the source of heterogeneity. Furthermore, a sensitivity analysis was conducted to examine the effect of variables on the pooled effect size. Funnel plots and Egger’s statistical tests were used to assess publication bias. A p value less than 0.05 was set to indicate statistical significance.

## Results

### Selection and identification of studies

**Figure 1:**
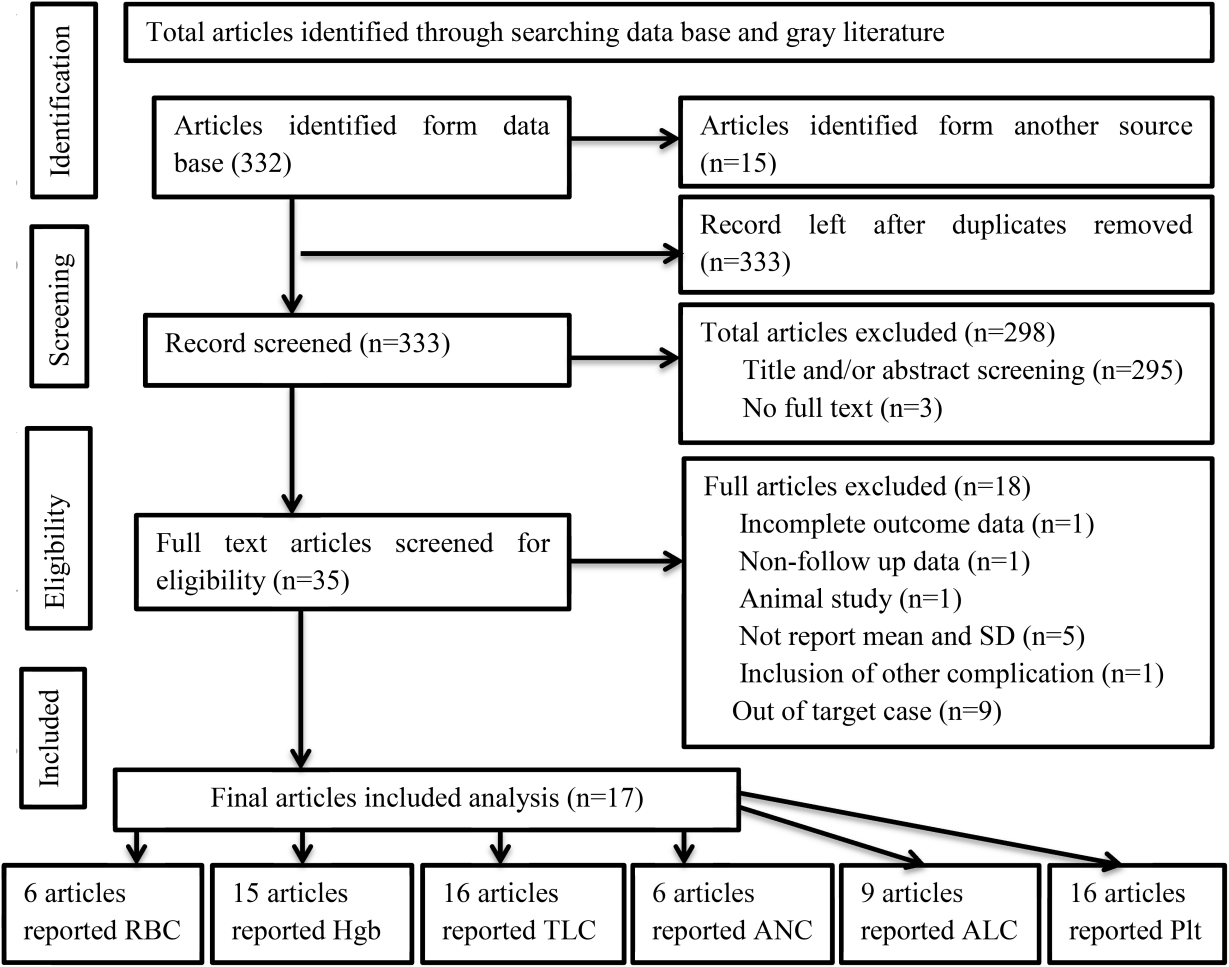
PRISMA flow diagram of article selection for systematic review and meta-analysis of the effect of RAI therapy on hematological parameters among thyroid cancer patients.

A total of 367 articles were identified and retrieved through searches. Then, 34 duplicate studies were removed. The titles and/or abstracts of the remaining studies were further reviewed for eligibility, and 289 studies were found to be not relevant for analysis. After further screening the remaining articles, a total of 17 articles met the inclusion criteria and were included in the analysis. The number of included studies varied on the basis of the type of hematological parameters. The frequencies of studies reporting the means (SD) of RBCs, Hgb, TLCs, ANCs, ALCs, and PLTs before and after treatment were 6, 15, 16, 6, 9, and 16, respectively (**Figure 1**).

### Characteristics of the included studies

A total of 17 studies that assessed the effects of RAI treatment on hematological parameters were included. The majority were from Turkey (six) and China (three). The numbers of patients included before and after RAI therapy were 4,112 and 3,766, respectively. The majority (14) of the included studies were retrospective follow-up. Only two studies were conducted before 2010, and the remaining studies were conducted after 2010. The total follow-up period of the studies used to assess changes in hematological parameters ranged from one week to five years. In addition, the frequency of measurement was not consistent among the studies (**Table 1**).

**Table 1:** Summary characteristics of the articles included in the systematic review and meta-analysis (n=17).

Authors	Year of publication	Study area	Study design	Sample size before	Sample size after	Histology of thyroid cancer	Stage of cancer	Last follow up period
Bikas et al. (40)	2016	USA	Retrospective follow up	152	36	All thyroid cancer	All	5years
De Keizer et al. (52)	2004	Netherlands	Prospective follow up	14	14	Papillary and follicular	I and II	3months
Demir et al. (18)	2023	Turkey	Retrospective Follow up	225	225	Papillary and follicular	All	5years
Duskin-Bitan et al. (46)	2019	Israel	Retrospective Follow up	122	122	Papillary and follicular	All	1year
Dong et al. (43)	2020	China	Retrospective follow up	91	91	Papillary	I and II	3years
Hu et al. (47)	2016	China	Retrospective Follow up	385	197	Papillary and follicular	All	3months
Molinaro et al. (48)	2009	Italy	Retrospective Follow up	206	206	All thyroid cancer	All	1year
Padovani et al. (53)	2014	USA	Retrospective Follow up	20	20	Papillary and follicular	All	1year
Prinsen et al. (54)	2015	Netherlands	Retrospective Follow up	331	331	Papillary and follicular	III and IV	5years
Rui et al. (36)	2021	China	Retrospective Follow up	542	542	All thyroid cancer	NA	6months
Sahutoglu et al. (34)	2024	Turkey	Retrospective Follow up	97	97	All thyroid cancer	NA	6months
Sengoz et al. (45)	2020	Turkey	Prospective follow up	31	31	All thyroid cancer	NA	6months
Sönmez et al. (37)	2021	Turkey	Retrospective Follow up	1389	1389	All thyroid cancer	I, II and IV	5years
Sönmez et al. (55)	2010	Turkey	Retrospective Follow up	164	164	All thyroid cancer	All	1year
Soyluoglu et al. (56)	2023	Turkey	Retrospective Follow up	130	88	All thyroid cancer	I and II	1year
Vrndic et al. (41)	2016	Serbia	Prospective follow up	24	24	Papillary and follicular	NA	1 week
Yi et al. (35)	2020	Korea	Retrospective Follow up	189	189	Papillary	All	1year

### Quality and heterogeneity

The quality of each study was evaluated using the JBI checklist, and only high-quality (scored >75%) studies were included to minimize bias (**Supplementary File 2**). The outcome variables were tested via both fixed and random effects models. Significant heterogeneity was observed. As a result, a random effects model was used to minimize heterogeneity. Finally, the outcome variables were measured via SMD.

### Pooled estimated mean differences in hematological parameters after RAI therapy in thyroid cancer patients

#### Effect of RAI therapy on RBCs and their parameters in thyroid cancer patients

**Figure 2:**
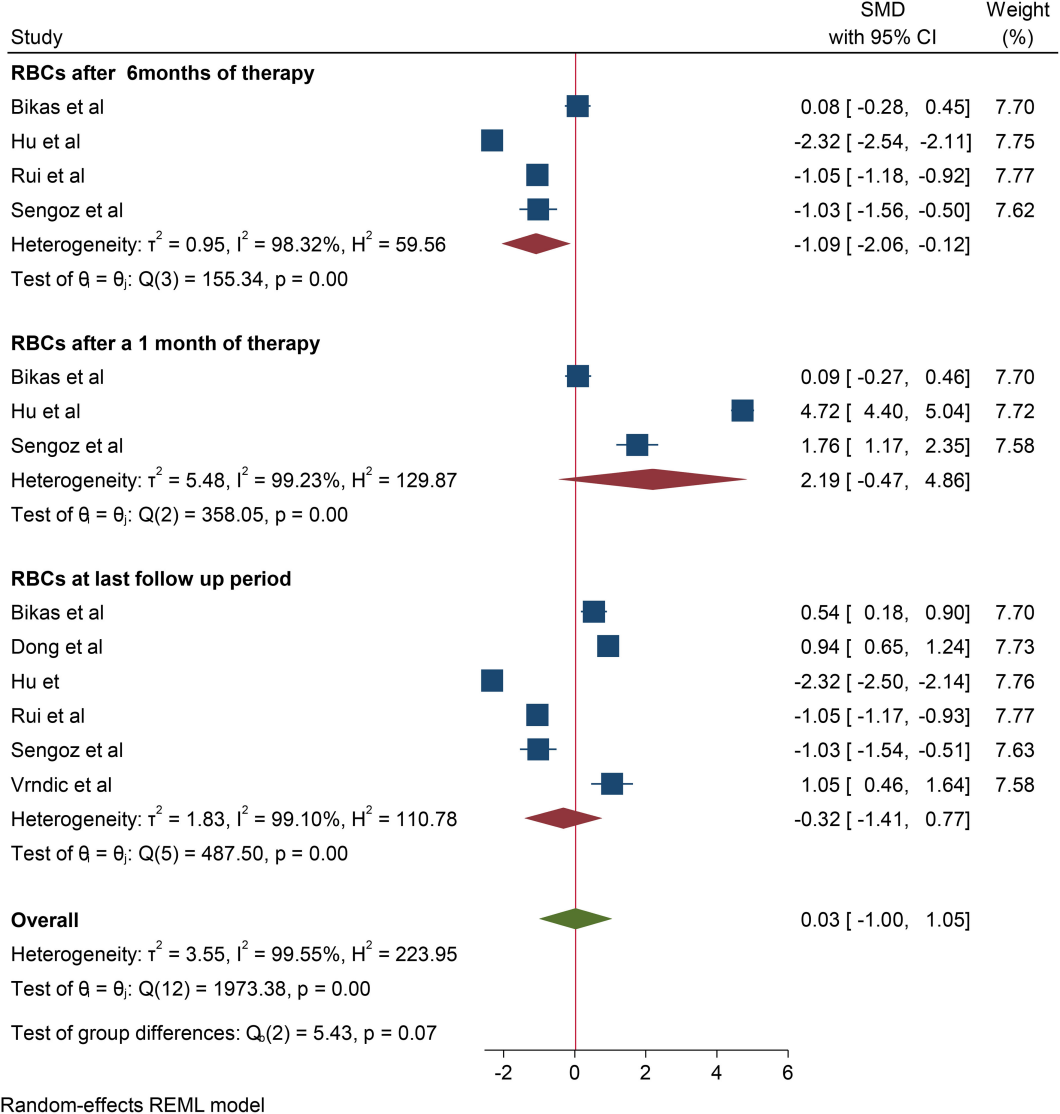
Forest plot depicting the effect of RIA therapy on RBC count at different time points.

A total of 6 studies were included to assess the effect of RAI therapy on RBC. Accordingly, the pooled SMD of the RBC count was -0.32*10^12^/L (95% CI: -1.35–0.71, p value: 0.56) at the last follow-up period. Four studies reported a change in the RBC count after 6 months of RAI therapy, and the change observed was -1.088*10^12^/L (95% CI: -1.96– -0.182, p value: 0.028). In addition, in three studies, the pooled SMD of RBC after one month of RAI therapy was 2.19*10^12^/L (95% CI: 0.93–03.53, p value: 0.106). No studies reported changes in RBC after 3 and 12 months of therapy (**Figure 2**).

**Figure 3:**
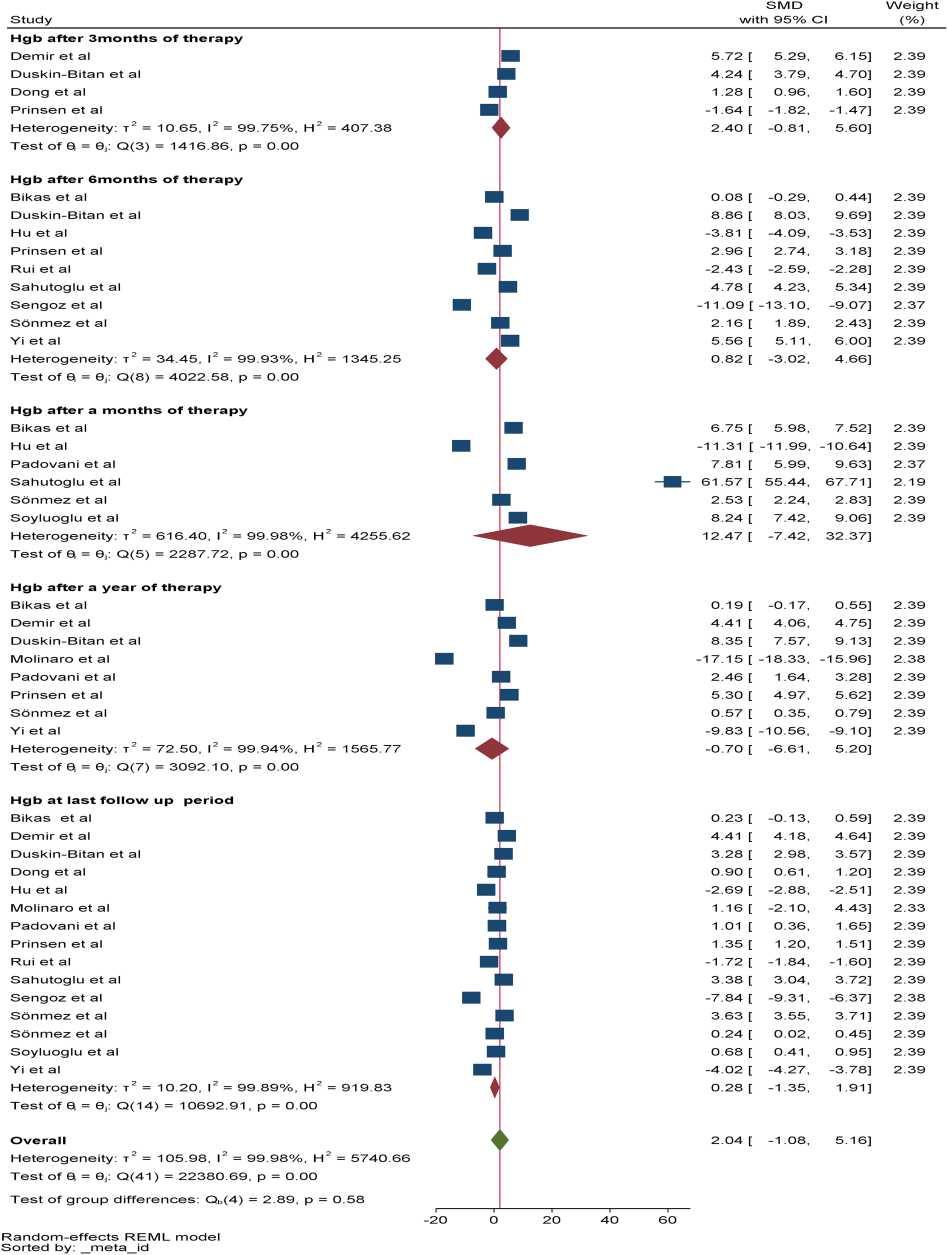
Forest plot depicting the effect of RIA therapy on RBC count at different time points.

Regarding the change in Hgb, a total of 15 studies were included in the meta-analysis to assess the overall effect of therapy on Hgb, and the overall effect size of Hgb at the last follow-up period was 0.28*g/dl (95% CI: -1.35-1.91, p value: 0.79). Among the included studies, six, four, and nine reported changes in Hgb after one, three, and six months of RAI therapy, respectively. The pooled SMDs of Hgb after one, three, and six months of RAI therapy were 12.47*g/dl (95% CI: -7.42-32.37, p value: 0.22), 2.4*g/dl (95% CI: -0.81-5.61, p value: 0.022) and 0.82*g/dl (95% CI: -3.02-4.66, p value: 0.68), respectively. In addition, eight studies reported a change in Hgb after one year of RAI therapy, and the pooled SMD of Hgb was -0.7*g/dl (95% CI: -6.6–5.2, p value: 0.82) (**Figure 3**).

#### Effect of RAI therapy on TLCs and their differential effects in thyroid cancer patients

**Figure 4:**
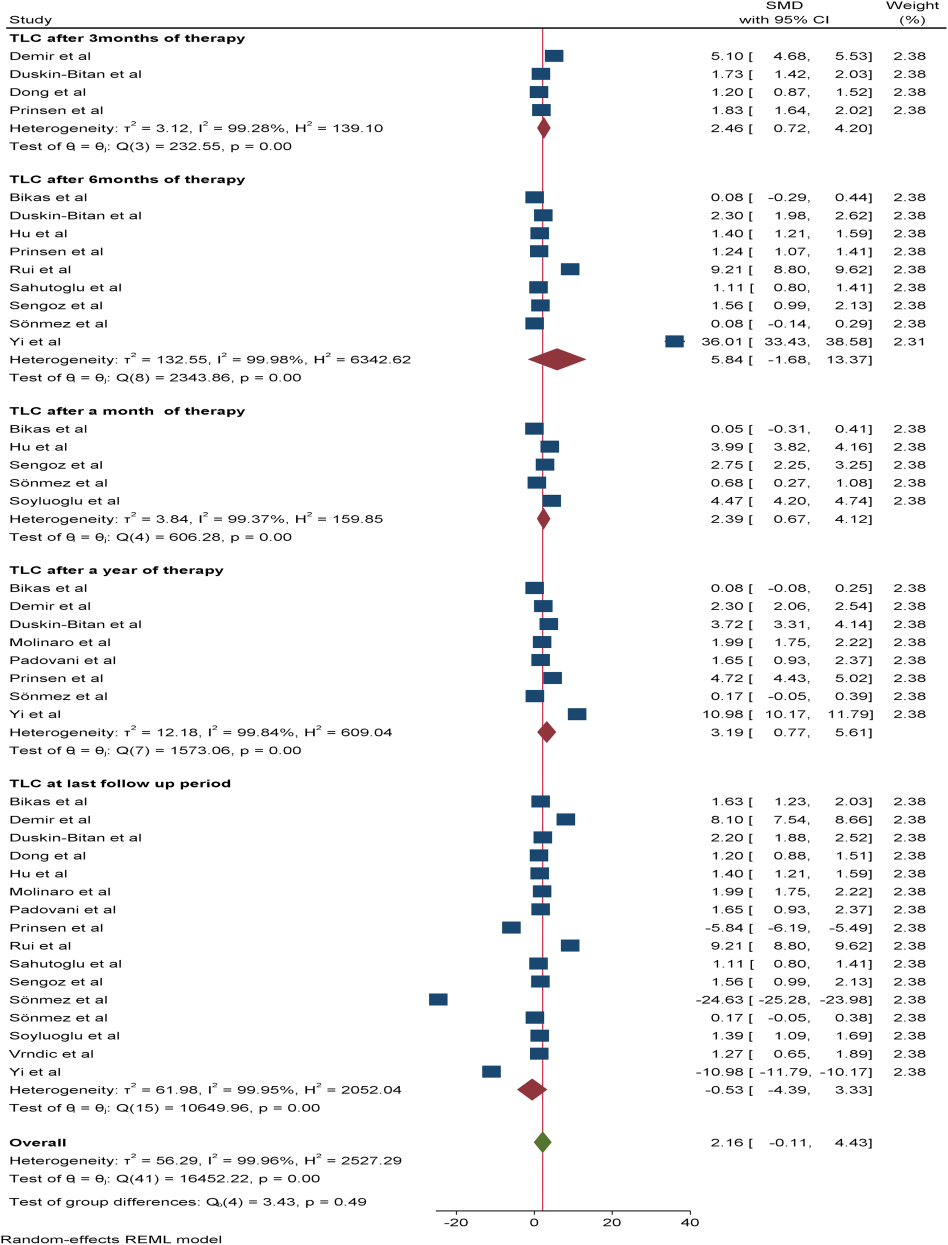
Forest plot depicting the TLCs after RAI therapy at different time points.

A total of 16 studies were included in the random effects model to assess the pooled effect of RAI therapy on TLC. Accordingly, the overall change in the mean TLC value after therapy was -0.53*10^9^/L (95% CI: -4.3-3.33, p value: 0.79). The pooled SMDs of TLC after one, three, six and 12 months of RAI therapy were 2.39*10^9^/L (95% CI: 0.67-4.12, p value: 0.007), 2.46*10^9^/L (95% CI: 0.72-4.2, p value: 0.006), 5.84*10^9^/L (95% CI: -1.68–13.37, p value: 0.013), and 3.19*10^9^/L (95% CI: 0.77–5.61, p value: 0.79), respectively (**Figure 4**).

**Figure 5:**
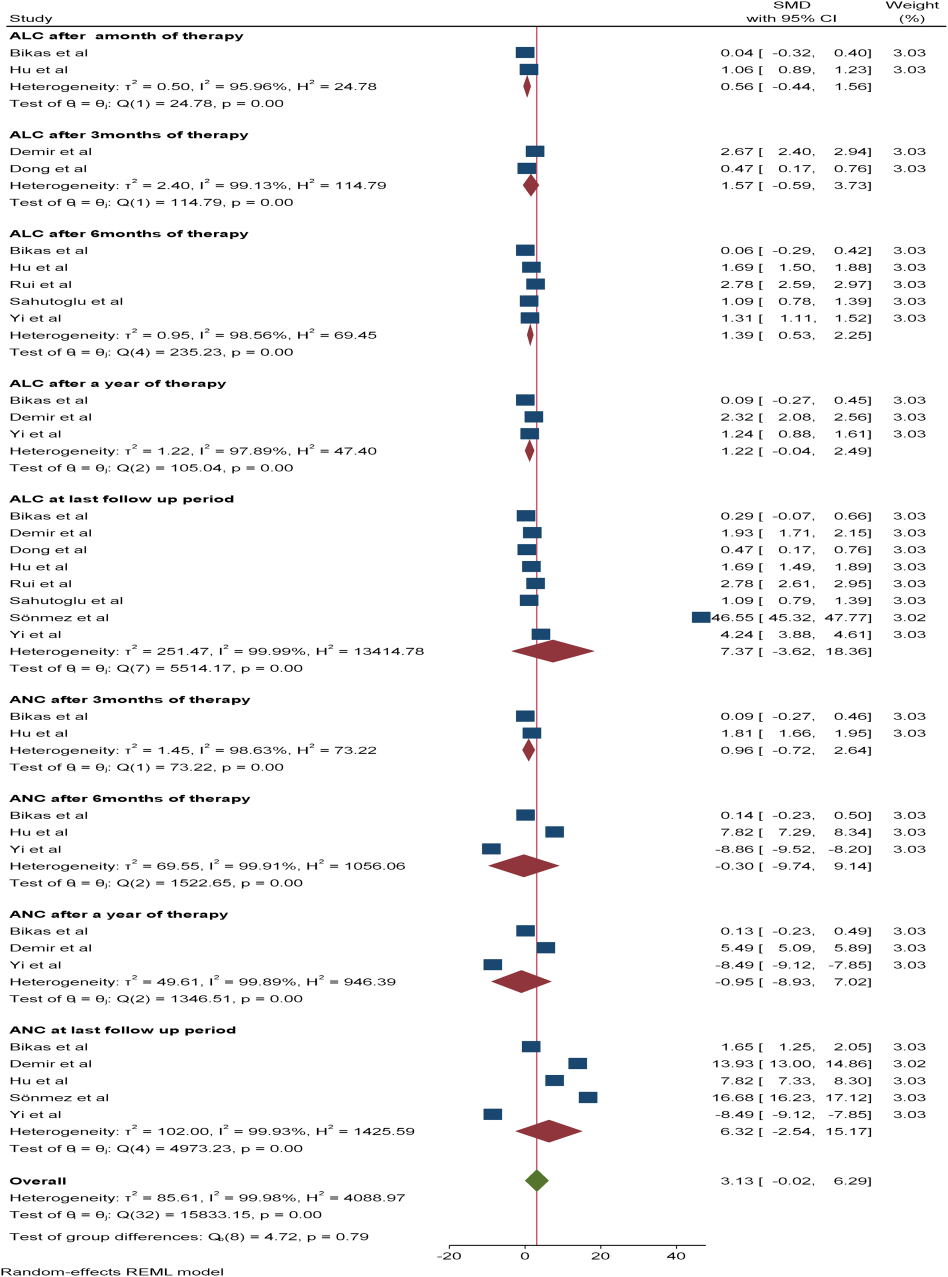
Forest plot depicting ANC and ALC after RAI therapy at different time points.

With respect to leucocyte differentials (ANC and ALC), six and nine studies, respectively, were included in the meta-analysis. The pooled SMDs of ANC and ALC at the last follow-up period were 6.32*10^9^/L (95% CI: -2.54-15.17) and 7.37 *10^9^/L (95% CI: -3.62-18.36), respectively, with a p value of <0.05. The SMDs of ANC and ALC after three, six, and 12 months of therapy were as follows: 1.57*10^9^/L (95% CI: -0.54-3.73, p value: 0.26) *vs* 0.96 *10^9^/L (95% CI: -0.74-2.64, p value: 0.15), 1.39*10^9^/L (95% CI: 0.53-2.25, p value: 0.95) *vs* -0.3*10^9^/L (95% CI: -9.74-9.14, p value: 0.0015) and 1.22*10^9^/L (95% CI: -0.04-2.49, p value: 0.79) *vs* -0.95*10^9^/L (95% CI: -8.93-7.02, p value: 0.0064), respectively. Additionally, the SMD of the ALC after one month of therapy was 0.56*10^9^/L (95% CI: -5.9-7.04, p value: 0.27) (**Figure 5**).

#### Effect of RAI therapy on PLTs in thyroid cancer patients

**Figure 6:**
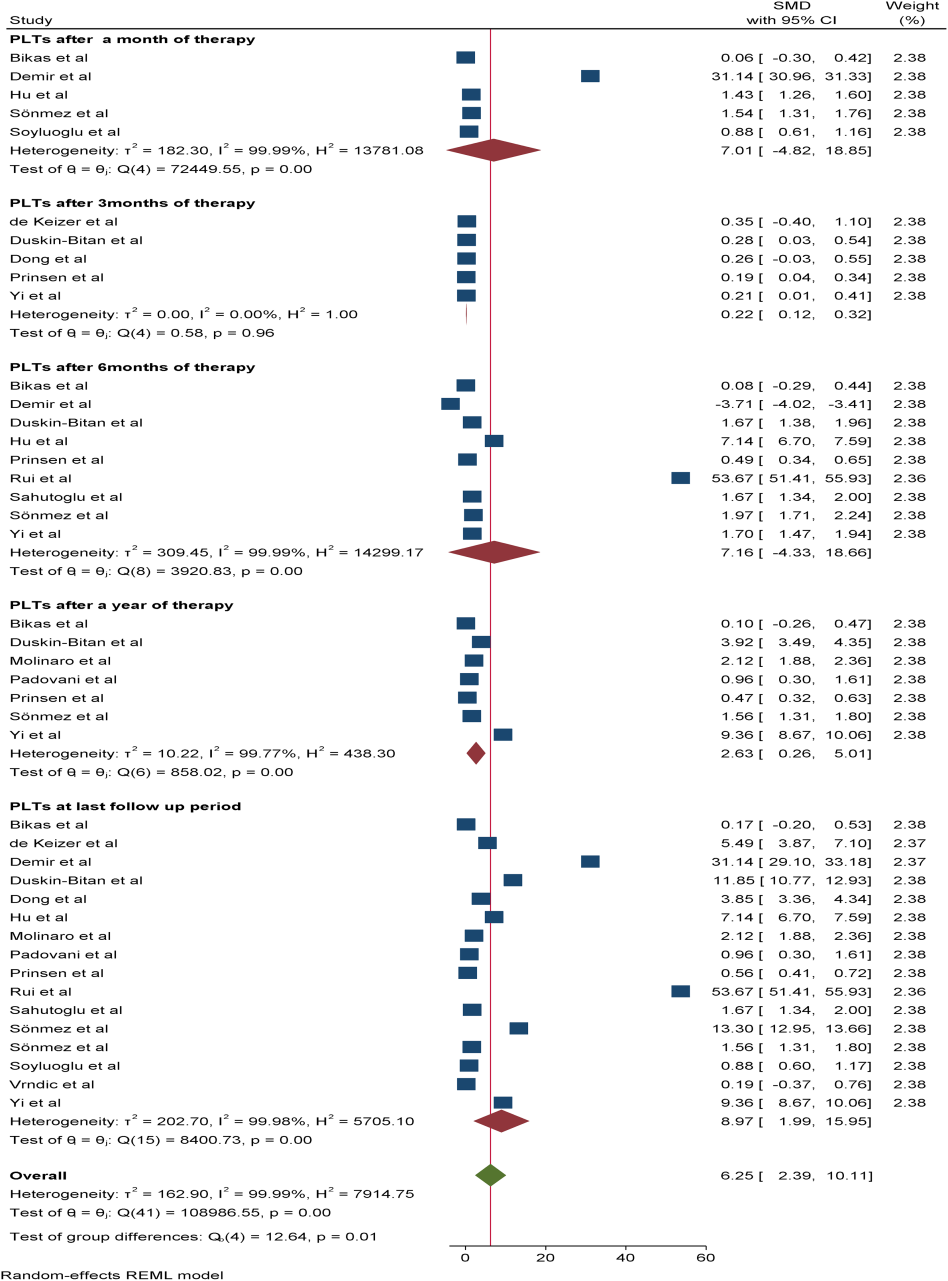
Forest plot depicting the PLT count after RAI therapy at different time points.

Changes in the peripheral PLT were reported in 16 studies. At the time of the last follow-up, the pooled SMD for the PLT count was 8.76*10^9^/L (95% CI: 6.56 - 10.96, p value: <0.001). Five of the included studies reported changes in the mean PLT count after one month (SMD: 7.01*10^9^/L, 95% CI: -6.56-20.56) and three months (SMD: 0.22*10^9^/L, 95% CI: 0.12-0.32) of therapy, with a p value <0.05. In addition, nine studies after 6 months and seven studies after one year of therapy reported a mean difference in the PLT count. Accordingly, the SMDs of the PLT count after six months and one year of therapy were 2.63*10^9^/L (95% CI: 1.26-3.93, p value: <0.001) and 6.61*10^9^/L (95% CI: 4.47-8.75, p value: <0.001), respectively (**Figure 6**).

### Subgroup analysis

According to the meta-analysis, significant heterogeneity (>75%) was observed in the case of all the hematological parameters included at different follow-up periods. As a result, subgroup analysis was performed on the basis of year of publication, continent, study design, histology of TC, and stage of TC to determine possible sources of heterogeneity. Accordingly, the subgroup analysis based on continent revealed that the pooled SMDs of the RBC count (*10^12^/L) and Hgb concentration (g/dl) in Asia, Europe, and North America were -0.81 *vs* -0.85, 0.01 *vs* 0.88 and 0.51 *vs* 0.51, respectively. Similarly, a subgroup analysis was conducted for TLC(*10^9^/L), ANC(*10^9^/L), and ALC(*10^9^/L), and the observed pooled SMDs in Asia were 0.61, 6.61 and 2.29; those in Europe were -1.65, 15.34 and 16.52; and those in North America were 1.64, 1.65 and 0.29, respectively. In terms of the PLT count (*10^9^/L), the pooled SMDs found in Asia, Europe, and North America were 17.15, 6.3 and 0.51, respectively. Similarly, the pooled SMDs of RBC and Hgb in Asia, Europe and North America were -0.8, 0.88, and 0.57 *vs* 2.28, 16.52, and 0.29, respectively.

The subgroup analysis conducted on the basis of the TC stage revealed that the SMDs of (RBC*10^9^/L, Hgb(*g/dl), TLC (*10^9^/L), ANC (*10^9^/L), ALC (*10^9^/L) and PLTs (*10^9^/L) of studies included patients with all stages of TC were -0.9, 0.35, 0.78, 3.72, 2.04, and 8.1, respectively. Meanwhile, the SMDs of Hgb, TLC, and PLTs in studies that included patients with only stage I and II disease were 0.79, 1.30, 0.79, and 3.32, respectively. With respect to subgroup analysis based on the histology of TC, the SMDs of RBC(*10^12^/L), Hgb(*g/dl), TLC(*10^9^/L), ANC(*10^9^/L), ALC(*10^9^/L) and PLTs(*10^9^/L) of studies that included all differentiated TC histologies were -0.51, -0.16, -0.95, 12.94, 12.67, and 10.46, respectively, whereas the SMDs of those parameters for studies that included only papillary and follicular TC histology were -0.12, 1.38, 1.42, 10.86, 1.37, and 7.62, respectively (**Supplementary File 3**).

### Sensitivity analysis

As a result of the high level of heterogeneity observed in the meta-analysis, sensitivity analysis was conducted to determine the impact of individual studies on the overall effect size. According to the analysis, omitting a single study at a time had no influence on the overall effect size or its significance for any of the included parameters (**Supplementary File 4**).

### Publication bias

Since the current meta-analysis was conducted to assess the effects of RAI therapy on hematological parameters at different periods of time, publication bias was specifically determined on the basis of the type of parameter and length of follow-up period (last follow-up, after one year, after the 6^th^ month, after the 3^rd^ month and after one month). Both visual inspection (funnel plot) and statistical tests (Egger’s test) were used to evaluate publication bias. The output of the analysis indicated that, except for Hgb after the 6^th^ month of RAI therapy, there was no publication bias among the studies included across parameters (**Supplementary File 5**).

### Trim and fill analysis

Significant publication bias was observed in the case of Hgb after the 6^th^ month of RAI therapy; as a result, trim-and-fill analysis was performed to minimize the effect of the small study. Accordingly, two additional studies were included in the model, and the overall effect size of Hgb after the 6^th^ month of RAI therapy in the random effects model was -0.776 (95% CI: -3.109-1.557, p value: 0.515).

## Discussion

Thyroid cancer is reported as a major public health concern globally (1, 2). Treating differentiated TC with radioactive iodine has been shown to have major side effects (12). The major side effects of RAI are hematopoietic system damage and subsequent dampening of peripheral blood cells (2, 14–16). Hematological side effects are associated with poor prognosis, increased risk of hospitalization, mortality and morbidity (34). Although single studies have reported the side effects of RAI on hematological parameters, the evidence is less clear and contradictory for patient management. Therefore, the main objective of this review was to assess the cumulative effects of RAI therapy on hematological parameters at different time points to assist in evidence-based patient management.

The findings of the meta-analysis revealed that TLC was significantly lower after one, three and six months of RAI therapy than at baseline. These findings indicate that the RAI has short- and intermediate-term effects on leucocytes. The possible explanations for the occurrence of leucopenia after RAI therapy could be related to organ toxicity, the activation of various inflammatory factors, alterations in growth factors and the induction of cellular apoptosis by RAI therapy (15, 16, 29).

Additionally, RAI induced alterations in genetic material and vital organs, which may also explain the decrease in TLC (15, 20, 21). Furthermore, the hematotoxic effect of therapy on hematopoietic precursor cells may be a possible reason for the decrease in TLC. On the other hand, a significant decrease in TLC was not observed after one year or at the last follow-up period. The lack of a significant decrease in the TCL after one year of RAI therapy may be due to recovery of the bone marrow and other hematopoietic organs, quick recovery capacity of the predominant total leucocytes (neutrophils), adaptive response of the body, and dose reduction over a period of time (15, 40). The lack of a significant decrease in the TLC at the last follow-up period can be attributed to the heterogeneity of the follow-up period.

Indeed, statistically significant decreases in ANC and ALC following RAI therapy were observed. This effect might be linked with treatment-induced organ toxicity and the activation of various inflammatory factors involved in changes in neutrophils and lymphocytes (15, 16, 29). In addition, a low ALC was observed after six and 12 months of therapy. The persistent low lymphocyte count could be explained by its radiosensitive nature, rapid apoptosis and lymphatic drainage compared with those of other TLCs (18, 41, 42).

In contrast, no significant change in ANC was observed after six or 12 months of therapy. This finding revealed that ANC returned to baseline after six months of therapy. The rapid recovery of neutrophils may be related to their quick regeneration capacity compared with those of other TLCs (18).Surprisingly, a decrease in the PLT count was observed at the last follow-up period and after one, three, six and 12 months of therapy compared with baseline. These findings suggest that RAI therapy has a persistent effect on the PLT. The possible reason for the persistent decrease in the PLT after therapy could be associated with vital organ damage, apoptosis, and genetic alterations (15, 20, 21). Another mechanism for the persistent decrease in the PLT count may be direct damage to megakaryocytes and their progenitors by radioisotopes (19).

With respect to RBC and Hgb, statistically significant changes were found after three and six months of therapy, respectively, compared to pre-treatment level. A possible explanation for the decreased RBC and Hgb after three and six months of therapy could be related to the timing of RBC maturation and bone marrow suppression; hence, the time taken for RBC maturation and bone marrow suppression is greater than 2 months after therapy (43–45). Although RIA therapy suppressed bone marrow, damaged other vital organs involved in erythropoiesis, and induced oxidative stress in RBCs, it had no short-term effect on RBC count or Hgb concentration (14, 19, 26, 27). During the other follow-up periods, the changes in RBC counts and Hgb counts were not statistically significant. This indicated that the effects of the RAI on RBCs and their parameters are minimal.

Significant heterogeneity was observed in the current meta-analysis. The possible explanations for high heterogeneity could be variability in sample size, electronic cell counters, the histology and the stage of TC, the target population and the study design. Accordingly, subgroup analysis was conducted to identify possible sources of heterogeneity. The subgroup analysis conducted on the basis of the TC stage revealed that the SMDs of Hgb, TLC, and PLTs were greater in studies that included patients with all stages of TC than in studies that included patients with only stages I and II. This may be related to the principle that more advanced disease is treated with higher activities of RAI. The observed high change in blood parameters in studies conducted with all stages may be related to the inclusion of advanced cases and does provided compared with stage I and II to activity (37, 46–48).

Subgroup analysis on the basis of the histology of TC also revealed that in studies that included all TC histology, the changes in Hgb, TLC, ANC, ALC, and PLT counts were greater after treatment than at baseline. This might be explained by variability in tumor biology; studies conducted in all stages may include more aggressive types of TC that induce the production of more proinflammatory factors (49). However, changes in TLC and Hgb were greater after RAI therapy in studies that included only papillary and follicular TC histology compared to those that included all histologies. This could also be attributed to variability in the avidity of RAI therapy depending on the type of TC. Classic subtypes of papillary TC, Follicular subtypes of papillary TC, and Follicular TC exhibit higher avidity, thereby prolonging the effective half-life of radioiodine, which could explain the more pronounced changes in hematological parameters (50, 51). Besides, the inclusion of all TC histologies may dilute the effect of RIA, leading to reduced changes in TLC and Hgb.

## Strengths and limitations

The current systematic review and meta-analysis was relatively comprehensive and assessed the effects of RAI therapy on hematological parameters at different periods of time, including major parameters. Additionally, articles were extensively searched, and only high-quality records were included in the meta-analysis. On the other hand, the majority of the studies included in this review were retrospective, which may affect the outcome and hinder the representativeness of the findings. Owing to a lack of data, some hematological parameters were not included in the analysis. Furthermore, owing to the variability in the RAI activity given to participants from the study, its effects on outcome variables were not assessed.

## Conclusion and recommendations

The total leucocyte count significantly decreased after one, three and six months of RAI therapy. Comparatively, the RAI was found to have a major effect on decreases in ANC and ALC. In addition, across all the follow-up periods included in the meta-analysis, a significant decrease in the PLT was observed. Furthermore, statistically significant changes in RBC and Hgb were observed only after three and six months of therapy, respectively. In general, this review revealed considerable changes in hematological parameters following RAI therapy in TC patients. These findings indicate that following RAI therapy, TC patients are at high risk for anemia, infection and bleeding. Thus, periodic screening and early management of hematological toxicity are very important to reduce further complications and improve the quality of life of patients.

## Data Availability

The original contributions presented in the study are included in the article/**Supplementary Material**. Further inquiries can be directed to the corresponding author.
