# Supplementary material for: Effect of radioactive iodine therapy on hematological parameters in patients with thyroid cancer: systematic review and meta-analysis
Source: Front Endocrinol (Lausanne). 2025 Mar 14;16:1562851. doi: 10.3389/fendo.2025.1562851 (PMC11950962; doi:10.3389/fendo.2025.1562851)
Supplement: Supplementary file 1 [file DataSheet1.docx]

Supplementary file 1: PubMed search strategy to assess changes in hematological parameters after radioiodine therapy among thyroid cancer patients

| Search | Search terms | Hits |
| --- | --- | --- |
| 1 | ((((((((((((((((((((((((((((((((((((((((((((((((((("Hematologicalparameter*"[Text Word]) OR ("hematological profil*"[Text Word])) OR ("Hematologic Test*"[Text Word])) OR ("hematological biomarker*"[Text Word])) OR ("biochemical marker*"[Text Word])) OR ("hematological finding*"[Text Word])) OR ("hematological abnormalit*"[Text Word])) OR ("blood Test*"[Text Word])) OR ("complete blood count"[Text Word])) OR (CBC[Text Word])) OR ("complete blood count parameter*"[Text Word])) OR ("peripheral blood parameter*"[Text Word])) OR ("Red blood cell count"[Text Word])) OR ("RBC count"[Text Word])) OR ("Erythrocyte Count"[Text Word])) OR ("RBC indice*"[Text Word])) OR ("Red blood cell parameter*"[Text Word])) OR ("RBC parameter*"[Text Word])) OR ("Erythrocyte parameter*"[Text Word])) OR (hemoglobin[Text Word])) OR (HB[Text Word])) OR (Hematocrit[Text Word])) OR (HCT[Text Word])) OR ("Mean cell volume"[Text Word])) OR ("mean corpuscular volume"[Text Word])) OR (MCV[Text Word])) OR ("mean cell hemoglobin"[Text Word])) OR ("mean corpuscular hemoglobin"[Text Word])) OR (MCH[Text Word])) OR ("Mean cell hemoglobin concentration"[Text Word])) OR ("mean corpuscular hemoglobin concentration"[Text Word])) OR (MCHC[Text Word])) OR ("Red blood cell distribution width"[Text Word])) OR ("RDW"[Text Word])) OR ("total white blood cell count"[Text Word])) OR (TWBC[Text Word])) OR ("white blood cell count"[Text Word])) OR ("absolute white blood cell count"[Text Word])) OR ("Leukocyte count"[Text Word])) OR ("total Leukocyte count"[Text Word])) OR ("absolute Leukocyte count"[Text Word])) OR ("absolute differential WBC count"[Text Word])) OR ("white blood cell differential count"[Text Word])) OR ("WBC differential count"[Text Word])) OR ("neutrophil count"[Text Word])) OR ("eosinophil count"[Text Word])) OR ("basophil count"[Text Word])) OR ("lymphocyte count"[Text Word])) OR ("monocyte count"[Text Word])) OR (plt[Text Word])) OR ("Platelet count*"[Text Word])) OR ("thrombocyte count"[Text Word]) | 532,039 |
| 2 | (((((((((((((((((((((((((((cancer[Text Word]) OR (tumo*[Text Word])) OR (malignanc*[Text Word])) OR (carcinoma[Text Word])) OR (adenocarcinoma*[Text Word])) OR (choriocarcinoma*[Text Word])) OR (sarcom*[Text Word])) OR (oncology[Text Word])) OR ("thyroid Cancer*"[Text Word])) OR ("thyroid neoplasm"[Text Word])) OR ("thyroid carcinoma"[Text Word])) OR ("thyroid Tumo*"[Text Word])) OR ("cancer of thyroid"[Text Word])) OR ("neoplasm of thyroid"[Text Word])) OR ("follicular thyroid cancer"[Text Word])) OR ("follicular thyroid tumo*"[Text Word])) OR ("follicular thyroid neoplasm"[Text Word])) OR ("papillary Thyroid Cancer"[Text Word])) OR ("papillary thyroid tumor"[Text Word])) OR ("papillary thyroid neoplasm"[Text Word])) OR ("anaplastic thyroid cancer"[Text Word])) OR ("anaplastic thyroid tumo*"[Text Word])) OR ("anaplastic thyroid neoplasm"[Text Word])) OR ("medullary thyroid cancer"[Text Word])) OR ("medullary thyroid tumo*"[Text Word])) OR ("medullary thyroid neoplasm"[Text Word])) OR ("nonmedullary thyroid cancer"[Text Word])) OR ("nonmedullary thyroid carcinom*"[Text Word]) | 4,495,881 |
| 3 | (((((((((((((((((("Thyroid therap*"[Text Word]) OR ("thyroid treatment*"[Text Word])) OR ("thyroid intervention"[Text Word])) OR ("iodine therapy"[Text Word])) OR ("iodine treatment"[Text Word])) OR ("Radioactive iodine therap*"[Text Word])) OR ("Radioactive iodine treatment"[Text Word])) OR ("RAI therapy"[Text Word])) OR ("RAI treatment"[Text Word])) OR ("Radioiodine therapy"[Text Word])) OR ("Radioiodine treatment"[Text Word])) OR (radioisotope[Text Word])) OR ("iodine radioisotope"[Text Word])) OR (isotope[Text Word])) OR (radioactive[Text Word])) OR ("Iodine-123 therapy"[Text Word])) OR ("I-123 therapy"[Text Word])) OR ("Iodine-131 therapy"[Text Word])) OR ("I-123 therapy"[Text Word]) | 199,251 |
|  | #1 and #2 and #3 | 302 |
