# Supplementary material for: Effect of radioactive iodine therapy on hematological parameters in patients with thyroid cancer: systematic review and meta-analysis
Source: Front Endocrinol (Lausanne). 2025 Mar 14;16:1562851. doi: 10.3389/fendo.2025.1562851 (PMC11950962; doi:10.3389/fendo.2025.1562851)
Supplement: Supplementary file 2 [file DataSheet2.docx]

### Supplementary file 2: Quality appraisal of the studies included in systematic review and meta-analysis to assess changes in hematological parameters after radioiodine therapy among thyroid cancer patients

Table1: The combined methodological quality of the each included studies using JBI critical appraising checklist (Includes question 1 to 6)

| Author | Q1 | Q2 | Q3 | Q4 | Q5 | | | | | | Q6 | | | | | |
| --- | --- | --- | --- | --- | --- | --- | --- | --- | --- | --- | --- | --- | --- | --- | --- | --- |
|  |  |  |  |  | O1 | O2 | O3 | O4 | O5 | O6 | O1 | O2 | O3 | O4 | O5 | O6 |
| Bikas et al (1) | Y | Y | Y | N | Y | Y | Y | Y | Y | Y | Y | Y | Y | Y | Y | Y |
| De Keizer et al (2) | Y | Y | Y | NA | NA | NA | NA | NA | NA | Y | NA | NA | NA | NA | NA | Y |
| Demir et al (3) | Y | Y | Y | N | NA | Y | Y | Y | Y | Y | NR | Y | Y | Y | Y | Y |
| Duskin-Bitan et al (4) | Y | Y | Y | N | NA | Y | Y | NA | NA | Y | NA | Y | Y | NA | NA | Y |
| Dong et al (5) | Y | Y | Y | N | Y | Y | Y | NA | Y | Y | Y | Y | Y | NA | Y | Y |
| Hu et al (6) | Y | Y | Y | N | Y | Y | Y | Y | Y | Y | Y | Y | Y | Y | Y | Y |
| Molinaro et al (7) | Y | Y | Y | N | NA | Y | Y | NA | NA | Y | NA | Y | Y | NA | NA | Y |
| Padovani et al (8) | Y | Y | Y | N | NA | Y | Y | NA | NA | Y | NA | Y | Y | NA | NA | Y |
| Prinsen et al (9) | Y | Y | Y | N | NA | Y | Y | NA | NA | Y | NA | Y | Y | NA | NA | Y |
| Rui et al (10) | Y | Y | Y | N | Y | Y | Y | Y | Y | Y | Y | Y | Y | Y | Y | Y |
| Sahutoglu et al (11) | Y | Y | Y | N | NA | Y | Y | NA | Y | Y | NA | Y | Y | NA | Y | Y |
| Sengoz et al (12) | Y | Y | Y | Y | Y | Y | Y | NA | NA | NA | Y | Y | Y | NA | NA | NA |
| Sönmez et al (13) | Y | Y | Y | N | NA | Y | Y | Y | Y | Y | NA | Y | Y | Y | Y | Y |
| Sönmez et al (14) | Y | Y | Y | N | NA | Y | Y | NA | NA | Y | NA | Y | Y | NA | NA | Y |
| Soyluoglu et al (15) | Y | Y | Y | N | NA | Y | Y | NA | NA | Y | NA | Y | Y | NA | NA | Y |
| Vrndic et al (16) | Y | Y | Y | UC | Y | NA | Y | NA | NA | Y | Y | NA | Y | NA | NA | Y |
| Yi et al (17) | Y | Y | Y | N | NA | Y | Y | Y | Y | Y | NA | Y | Y | Y | Y | Y |

Supplementary file 2 Continued …………………………………

Table2: The combined methodological quality of the each included studies using JBI critical appraising checklist (Includes question 7 to 9)

| Author | Q7 | | | | | | Q8 | | | | | | Q9 | | | | | | Overall quality |
| --- | --- | --- | --- | --- | --- | --- | --- | --- | --- | --- | --- | --- | --- | --- | --- | --- | --- | --- | --- |
|  | O1 | O2 | O3 | O4 | O5 | O6 | O1 | O2 | O3 | O4 | O5 | O6 | O1 | O2 | O3 | O4 | O5 | O6 | High |
| Bikas et al (1) | Y | Y | Y | Y | Y | Y | Y | Y | Y | Y | Y | Y | Y | Y | Y | Y | Y | Y |  |
| De Keizer et al (2) | NA | NA | NA | NA | NA | Y | NA | NA | NA | NA | NA | Y | NA | NA | NA | NA | NA | Y | High |
| Demir et al (3) | NR | Y | Y | Y | Y | Y | N | Y | Y | Y | Y | Y | N | Y | Y | Y | Y | Y | High |
| Duskin-Bitan et al (4) | NA | Y | Y | NA | NA | Y | NA | Y | Y | NA | NA | Y | NA | Y | Y | NA | NA | Y | High |
| Dong et al (5) | Y | Y | Y | NA | Y | Y | Y | Y | Y | NA | Y | Y | Y | Y | Y | NA | Y | Y | High |
| Hu et al (6) | Y | Y | Y | Y | Y | Y | Y | Y | Y | Y | Y | Y | Y | Y | Y | Y | Y | Y | High |
| Molinaro et al (7) | NA | Y | Y | NA | NA | Y | NA | Y | Y | NA | NA | Y | NA | Y | Y | NA | NA | Y | High |
| Padovani et al (8) | NA | Y | Y | NA | NA | Y | NA | Y | Y | NA | NA | Y | NA | Y | Y | NA | NA | Y | High |
| Prinsen et al (9) | NA | Y | Y | NA | NA | Y | NA | Y | Y | NA | NA | Y | NA | Y | Y | NA | NA | Y | High |
| Rui et al (10) | Y | Y | Y | Y | Y | Y | Y | Y | Y | Y | Y | Y | Y | Y | Y | Y | Y | Y | High |
| Sahutoglu et al (11) | NA | Y | Y | NA | Y | Y | NA | Y | Y | NA | Y | Y | NA | Y | Y | NA | Y | Y | High |
| Sengoz et al (12) | Y | Y | Y | NA | NA | NA | Y | Y | Y | NA | NA | NA | Y | Y | Y | NA | NA | NA | High |
| Sönmez et al (13) | NA | Y | Y | Y | Y | Y | NA | Y | Y | Y | Y | Y | NA | Y | Y | Y | Y | Y | High |
| Sönmez et al (14) | NA | Y | Y | NA | NA | Y | NA | Y | Y | NA | NA | Y | NA | Y | Y | NA | NA | Y | High |
| Soyluoglu et al (15) | NA | Y | Y | NA | NA | Y | NA | Y | Y | NA | NA | Y | NA | Y | Y | NA | NA | Y | High |
| Vrndic et al (16) | Y | NA | Y | NA | NA | Y | Y | NA | Y | NA | NA | Y | Y | NA | Y | NA | NA | Y | High |
| Yi et al (17) | NA | Y | Y | Y | Y | Y | NA | Y | Y | Y | Y | Y | NA | Y | Y | Y | Y | Y | High |

**Note. High quality** (75-100 %) score**, Y**= Yes; **N**= Not reported, **UC**=Unclear, **NA**=not applicable, **O1**=outcome 1(RBC), **O2**=outcome 2(Hgb), **O3**=outcome 3 (TLC), **O4**=outcome 4(ANC), **O5**=outcome 5(ALC), **O6**=outcome 6(PLT).

**Question codes**

Q1. Is it clear in the study what is the “cause” and what is the “effect” (i.e. there is no confusion about which variable comes first)?

Q2. Was there a control group?

Q3. Were participants included in any comparisons similar?

Q4. Were the participants included in any comparisons receiving similar treatment/care, other than the exposure or intervention of interest?

Q5. Were there multiple measurements of the outcome, both pre and post the intervention/exposure?

Q6. Were the outcomes of participants included in any comparisons measured in the same way?

Q7. Were outcomes measured in a reliable way?

Q8. Was follow-up complete and if not, were differences between groups in terms of their follow-up adequately described and analyzed?

Q9. Was appropriate statistical analysis used?

**References**

1. Bikas A, Schneider M, Desale S, Atkins F, Mete M, Burman KD, et al. Effects of dosimetrically guided I-131 therapy on hematopoiesis in patients with differentiated thyroid cancer. The Journal of Clinical Endocrinology & Metabolism. 2016;101(4):1762-9.

2. **de Keizer B**, Hoekstra A, Konijnenberg MW, de Vos F, Lambert B, van Rijk PP, et al. Bone marrow dosimetry and safety of high 131I activities given after recombinant human thyroid-stimulating hormone to treat metastatic differentiated thyroid cancer. Journal of nuclear medicine : official publication, Society of Nuclear Medicine. 2004;45(9):1549-54.

3. Demir AN, Kara Z, Sulu C, Uysal S, Zulfaliyeva G, Atar OA, et al. The effect of radioiodine therapy on blood cell count in patients with differentiated thyroid cancer. Hormones. 2023;22(4):595-602.

4. Duskin-Bitan H, Leibner A, Amitai O, Diker-Cohen T, Hirsch D, Benbassat C, et al. Bone-marrow suppression in elderly patients following empiric radioiodine therapy: real-life data. Thyroid : official journal of the American Thyroid Association. 2019;29(5):683-91.

5. Dong P, Wang L, Huang R, Li L. Bone marrow suppression in pediatric patients with differentiated thyroid cancer following empirical radioiodine therapy. Medicine. 2020;99(31):e21398.

6. Hu T, Meng Z, Zhang G, Jia Q, Tan J, Zheng W, et al. Influence of the first radioactive iodine ablation on peripheral complete blood count in patients with differentiated thyroid cancer. Medicine. 2016;95(35):e4451.

7. Molinaro E, Leboeuf R, Shue B, Martorella AJ, Fleisher M, Larson S, et al. Mild decreases in white blood cell and platelet counts are present one year after radioactive iodine remnant ablation. Thyroid : official journal of the American Thyroid Association. 2009;19(10):1035-41.

8. Padovani RP, Tuttle RM, Grewal R, Larson SM, Boucai L. Complete blood counts are frequently abnormal 1 year after dosimetry-guided radioactive iodine therapy for metastatic thyroid cancer. Endocrine practice : official journal of the American College of Endocrinology and the American Association of Clinical Endocrinologists. 2014;20(3):213-20.

9. Prinsen HT, Klein Hesselink EN, Brouwers AH, Plukker JT, Sluiter WJ, van der Horst-Schrivers AN, et al. Bone marrow function after 131I therapy in patients with differentiated thyroid carcinoma. The Journal of Clinical Endocrinology & Metabolism. 2015;100(10):3911-7.

10. Rui Z, Wu R, Zheng W, Wang X, Meng Z, Tan J. Effect of 131I therapy on complete blood count in patients with differentiated thyroid cancer. Medical science monitor: international medical journal of experimental and clinical research. 2021;27:e929590-1.

11. Sahutoglu G, ÇETİN S, Atilgan H. The Effect of I-131 Treatment on Complete Blood Count. Atom Indonesia. 2024;50(1).

12. Sengoz T, Kilic-Toprak E, Yaylali O, Kilic-Erkek O, Ozdemir Y, Oymak B, et al. Hemorheology and oxidative stress in patients with differentiated thyroid cancer following I-131 ablation/metastasis treatment. Clinical Hemorheology and Microcirculation. 2020;74(2):209-21.

13. Sönmez B, Bektaş Ö, Erkut N, Sönmez M. Assessment of Long-Term Hematologic Effects in Differentiated Thyroid Cancer Patients Treated with Radioactive Iodine. Turkish Journal of Hematology. 2021;38(4):306.

14. Sönmez B, Doğan İ, Yavruoğlu C, Can G, Sönmez M. Radyoaktif iyot tedavisi alan tiroid kanserli hastalarda tam kan sayımı değişiklikleri. 2010.

15. Soyluoglu S, Andac B, Korkmaz U, Ustun F. Assessment of three different radioiodine doses for ablation therapy of thyroid remnants: Efficiency, complications and patient comfort. Medicine. 2023;102(39):e35339.

16. Vrndic OB, Djurdjevic PM, Jovanovic DD, Mijatovic Teodorovic LC, Kostic IR, Jeftic ID, et al. Blood cells in thyroid cancer patients: a possible influence of apoptosis. Open Medicine. 2016;11(1):87-92.

17. Yi W, Kim BH, Kim M, Ryang SR, Jang MH, Kim JM, et al. Short-term bone marrow suppression in differentiated thyroid cancer patients after radioactive iodine treatment. Endocrine journal. 2020;67(12):1193-8.
