# Supplementary material for: Effect of radioactive iodine therapy on hematological parameters in patients with thyroid cancer: systematic review and meta-analysis
Source: Front Endocrinol (Lausanne). 2025 Mar 14;16:1562851. doi: 10.3389/fendo.2025.1562851 (PMC11950962; doi:10.3389/fendo.2025.1562851)
Supplement: Supplementary file 3 [file DataSheet3.docx]

**Supplementary file 3: Sub-group analysis**

Figure A: A Sub-group analysis of RBCs count based on study design, continent, histology and stage of thyroid cancer

Figure B: A Sub-group analysis of Hgb based on study design, continent, histology and stage of thyroid cancer

**** Figure C: A Sub-group analysis of TLC count based on study design, continent, histology and stage of thyroid cancer

Figure D: A Sub-group analysis of ANCs based on study design, continent, histology and stage of thyroid cancer

Figure E: A Sub-group analysis of ALC based on study design, continent, histology and stage of thyroid cancer

Figure F: A Sub-group analysis of PLT count based on study design, continent, histology and stage of thyroid cancer
