# Supplementary material for: Effect of radioactive iodine therapy on hematological parameters in patients with thyroid cancer: systematic review and meta-analysis
Source: Front Endocrinol (Lausanne). 2025 Mar 14;16:1562851. doi: 10.3389/fendo.2025.1562851 (PMC11950962; doi:10.3389/fendo.2025.1562851)
Supplement: Supplementary file 4 [file DataSheet4.docx]

**Supplementary file 4 Sensitivity analysis**

Table 1: Sensitivity analysis to estimate the effect each study on the RBCs count among thyroid cancer patients receiving radioiodine therapy.

| Omitted study | SMD | 95%CI | p-Value |
| --- | --- | --- | --- |
| Bikas et al | -0.492 | -1.764-0.77 | 0.448 |
| Dong et al | -0.576 | -1.76-0.61 | 0.342 |
| Hu et al | 0.084 | -0.83-1.004 | 0.858 |
| Rui et al | -0.170 | -1.46-1.124 | 0.796 |
| Sengoz et al | -0.180 | -1.473-1.113 | 0.785 |
| Vrndic et al | -0.587 | -1.755-0.581 | 0.325 |
| Combined | -0.321 | -1.414-0.772 |  |

Table 2: Sensitivity analysis to estimate the effect each study on the Hgb among thyroid cancer patients receiving radioiodine therapy.

| Omitted study | SMD | 95%CI | p-Value |
| --- | --- | --- | --- |
| Bikas et al | 0.227 | -1.641-2.096 | 0.811 |
| Demir et al | -0.092 | -1.828-1.645 | 0.918 |
| Duskin-Bitan, et al | -0.006 | -1.806-1.794 | 0.995 |
| Dong et al | 0.175 | -1.690-2.041 | 0.854 |
| Hu et al | 0.455 | -1.348-2.259 | 0.621 |
| Padovani et al | 0.168 | -1.696-2.032 | 0.860 |
| Prinsen et al | 0.141 | -1.719-2.001 | 0.882 |
| Rui et al | 0.379 | -1.461-2.219 | 0.686 |
| Sahutoglu et al | -0.014 | -1.809-1.782 | 0.988 |
| Sengoz et al | 0.821 | -0.556-2.197 | 0.243 |
| Sönmez et al | -0.033 | -1.816-1.749 | 0.971 |
| Sönmez et al | 0.227 | -1.642-2.096 | 0.812 |
| Soyluoglu et al | 0.193 | -1.675-2.060 | 0.840 |
| Yi et al | 0.560 | -1.167-2.288 | 0.525 |
| Combined | 0.230 | -1.498-1.957- | 0.794 |

Table 3: Sensitivity analysis to estimate the effect each study on the TLC among thyroid cancer patients receiving radioiodine therapy.

| Omitted study | SMD | 95%CI | P-value |
| --- | --- | --- | --- |
| Bikas et al | -0.678 | -4.793-3.437 | 0.747 |
| Demir et al | -1.109 | -5.054-2.837 | 0.582 |
| Duskin-Bitan et al | -0.716 | -4.824-3.393 | 0.733 |
| Dong et al | -0.649 | -4.768-3.471 | 0.758 |
| Hu et al | -0.662 | -4.780-3.455 | 0.753 |
| Molinaro et al | -0.702 | -4.813-3.410 | 0.738 |
| Padovani et al | -0.679 | -4.794-3.436 | 0.746 |
| Prinsen et al | -0.179 | -4.238­-3.880 | 0.931 |
| Rui et al | -1.183 | -5.078-2.712 | 0.552 |
| Sahutoglu et al | -0.643 | -4.763-3.477 | 0.760 |
| Sengoz et al | -0.673 | -4.789-3.443 | 0.749 |
| Sönmez et al | 1.074 | -1.312-3.460 | 0.378 |
| Sönmez et al | -0.580 | -4.705-3.545 | 0.783 |
| Soyluoglu et al | -0.662 | -4.779-3.456 | 0.753 |
| Vrndic et al | -0.654 | -4.772-3.464 | 0.756 |
| Yi et al | 0.162 | -3.698-4.022 | 0.935 |
| Combined | -0.533 | -4.393-3.326 | 0.787 |

Table 4: Sensitivity analysis to estimate the effect each study on the ANC among thyroid cancer patients receiving radioiodine therapy.

| Omitted study | SMD | [95% conf. | P- value |
| --- | --- | --- | --- |
| Bikas et al | 10.087 | 0.123-20.050 | 0.047 |
| Demir et al | 7.631 | -2.580-17.843 | 0.143 |
| Hu et al | 8.853 | -1.659-19.365 | 0.099 |
| Rui et al | 6.316 | -2.540-15.173 | 0.162 |
| Sönmez et al | 7.079 | -2.714-16.871 | 0.157 |
| Yi et al | 12.110 | 5.570-18.649 | 0.000 |
| Combined | 8.679 | 0.091-17.268 | 0.048 |

Table 5: Sensitivity analysis to estimate the effect each study on the ALC among thyroid cancer patients receiving radioiodine therapy.

| Omitted study | SMD | [95% conf. | P value |
| --- | --- | --- | --- |
| Bikas et al | 8.385 | -4.100-20.869 | 0.188 |
| Demir et al | 8.151 | -4.419-20.721 | 0.204 |
| Dong et al | 8.360 | -4.135-20.855 | 0.190 |
| Hu et al | 8.186 | -4.373-20.744 | 0.201 |
| Rui et al | 8.030 | -4.576-20.635 | 0.212 |
| Sahutoglu et al | 8.272 | -4.257-2.806 | 0.196 |
| Sönmez et al | 1.785 | 0.764-2.806 | 0.001 |
| Yi et al | 7.821 | -4.831-20.473 | 0.226 |
| Combined | 7.372 | -3.618-18.363 | 0.189 |

Table 6: Sensitivity analysis to estimate the effect each study on the PLT among thyroid cancer patients receiving radioiodine therapy.

| Omitted | SMD | 95%CI | p- value |
| --- | --- | --- | --- |
| Bikas et al | 9.559 | 2.196-16.921 | 0.011 |
| de Keizer et al | 9.204 | 1.756-16.651 | 0.015 |
| Demir et al | 7.496 | 0.708-14.284 | 0.030 |
| Duskin-Bitan et al | 8.780 | 1.327-16.233 | 0.021 |
| Dong et al | 9.314 | 1.883-16.744 | 0.014 |
| Hu et al | 9.094 | 1.634-16.554 | 0.017 |
| Molinaro et al | 9.429 | 2.025-16.832 | 0.013 |
| Padovani et al | 9.506 | 2.126-16.886 | 0.012 |
| Prinsen et al | 9.532 | 2.161-16.904 | 0.011 |
| Rui et al | 5.989 | 1.879-10.099 | 0.004 |
| Sahutoglu | 9.459 | 2.064-16.853 | 0.012 |
| Sönmez et al | 8.682 | 1.243-16.122 | 0.022 |
| Sönmez et al | 9.466 | 2.074-16.859 | 0.012 |
| Soyluoglu | 9.511 | 2.132-16.889 | 0.012 |
| Vrndic et al | 9.557 | 2.194-16.920 | 0.011 |
| Yi et al | 8.946 | 1.481-16.410 | 0.019 |
| Combined | 8.970 | 1.990-15.950 | 0.012 |
