# Supplementary material for: Effect of radioactive iodine therapy on hematological parameters in patients with thyroid cancer: systematic review and meta-analysis
Source: Front Endocrinol (Lausanne). 2025 Mar 14;16:1562851. doi: 10.3389/fendo.2025.1562851 (PMC11950962; doi:10.3389/fendo.2025.1562851)
Supplement: Supplementary file 5 [file DataSheet5.docx]

**Supplementary file 5: Publication bias analysis**

Funnel plot of included studies for TLC. NB: A. effect of RAI on TLC at last follow up of TC patients, B. effect of RAI on TLC after a year of TC patients, C. effect of RAI on TLC after 6months of TC patients, D. effect of RAI on TLC after 3months of TC patients, E. effect of RAI on TLC after a months of TC patients.

Funnel plot of included studies for PLTs. NB: A. effect of RAI on PLTs at last follow up of TC patients, B. effect of RAI on PLTs after a year of TC patients, C. effect of RAI on PLTs after 6months of TC patients, D. effect of RAI on PLTs after 3months of TC patients, E. effect of RAI on PLTs after a months of TC patients.

Funnel plot of included studies for Hgb NB: A. effect of RAI on PLTs at last follow up of TC patients, B. effect of RAI on PLTs after a year of TC patients, C. effect of RAI on PLTs after 6months of TC patients, D. effect of RAI on PLTs after 3months of TC patients, E. effect of RAI on PLTs after a months of TC patients.

Funnel plot of included studies for ALC NB: A. effect of RAI on ALC at last follow up of TC patients, B. effect of RAI on ALC after a year of TC patients, C. effect of RAI on ALC after 6months of TC patients, D. effect of RAI on ALC after 3months of TC patients, E. effect of RAI on ALC after a months of TC patients.

Funnel plot of included studies for ANC NB: A. effect of RAI on ANC at last follow up of TC patients, B. effect of RAI on ANC after a year of TC patients, C. effect of RAI on ANC after 6months of TC patients, D. effect of RAI on ALC after a month of TC patients

Funnel plot of included studies for RBCs NB: A. effect of RAI on RBCs at last follow up of TC patients, B. effect of RAI on RBCs after 6months of TC patients, C. effect of RAI on RBCs after 1month of TC patients
